# Dapagliflozin *versus* sacubitril–valsartan for heart failure with mildly reduced or preserved ejection fraction

**DOI:** 10.3389/fphar.2024.1357673

**Published:** 2024-03-19

**Authors:** Ronen Arbel, Abed N. Azab, Mansi Oberoi, Enis Aboalhasan, Artyom Star, Khaled Elhaj, Fouad Khalil, Hilmi Alnsasra

**Affiliations:** ^1^ Maximizing Health Outcomes Research Lab, Sapir College, Ashkelon, Israel; ^2^ Department of Cardiology, Soroka University Medical Center, Beersheba, Israel; ^3^ Department of Nursing, Department of Clinical Biochemistry and Pharmacology, Faculty of Health Sciences, Ben Gurion University of the Negev, Be’er Sheva, Israel; ^4^ University of Nebraska Medical Center, Omaha, NE, United States; ^5^ Faculty of Health Sciences, Ben Gurion University of the Negev, Be’er Sheva, Israel

**Keywords:** heart failure with mildly reduced or preserved ejection fraction, dapagliflozin, sacubitril–valsartan, cost needed to treat, outcomes, SLGT-2, neprilysin

## Abstract

**Background and aim:**

Heart failure with preserved ejection fraction (HFpEF) is associated with an increased risk of heart failure (HF) hospitalizations and cardiovascular death (CVD). Both dapagliflozin and sacubitril–valsartan have recently shown convincing reductions in the combined risk of CVD and HF hospitalizations in patients with HF and mildly reduced ejection fraction (HFmrEF) or HFpEF. We aimed to investigate the cost-per-outcome implications of dapagliflozin vs sacubitril–valsartan in the treatment of HFmrEF or HFpEF patients.

**Methods:**

We compared the annualized cost needed to treat (CNT) to prevent the composite outcome of total HF hospitalizations and CVD with dapagliflozin or sacubitril–valsartan. The CNT was estimated by multiplying the annualized number needed to treat (aNNT) by the annual cost of therapy. The aNNT was calculated based on data collected from the DELIVER trial for dapagliflozin and a pooled analysis of the PARAGLIDE-HF and PARAGON-HF trials for sacubitril–valsartan. Costs were based on 2022 US prices. Scenario analyses were performed to attenuate the differences in the studies’ populations.

**Results:**

The aNNT with dapagliflozin in DELIVER was 30 (95% confidence interval [CI]: 21-62) *versus* 44 (95% CI: 25-311) with sacubitril–valsartan in a pooled analysis of PARAGLIDE-HF and PARAGON-HF, with an annual cost of $4,951 and $5,576, respectively. The corresponding CNTs were $148,547.13 (95% CI: $103,982.99–$306,997.39) for dapagliflozin and $245,346.77 (95% CI: $139,401.58–1,734,155.60) for sacubitril–valsartan for preventing the composite outcome of CVD and HF hospitalizations. The CNT for preventing all-cause mortality was lower for dapagliflozin than sacubitril–valsartan $1,128,958.15 [CI: $401,077.24–∞] vs $2,185,816.71 [CI: $607,790.87–∞].

**Conclusion:**

Dapagliflozin provides a better monetary value than sacubitril–valsartan in preventing the composite outcome of total HF hospitalizations and CVD among patients with HFmrEF or HFpEF.

## 1 Introduction

Nearly half of all cases of heart failure (HF) in the United States are caused by HF with a preserved ejection fraction (HFpEF) (Tsao et al., 2023). By 2030, HF is expected to account for 69.8 billion dollars of the annual healthcare cost in the United States (Tsao et al., 2023). As the population ages, it is predicted that HFpEF will continue to become a more important public health issue (Tsao et al., 2023).

There is well-established evidence that sodium–glucose transporter 2 inhibitors (SGLT2Is) and angiotensin receptor/neprilysin inhibitors (ARNIs) are effective treatments for HF with a reduced ejection fraction (HFrEF) (McMurray et al., 2014; 2019; Packer et al., 2020). However, in HFpEF patients, spironolactone and sacubitril–valsartan have only shown marginal benefits (Pitt et al., 2014; McMurray et al., 2020). Recently, dapagliflozin demonstrated the most convincing reduction in adverse outcomes in HFpEF patients (Solomon et al., 2022).

Dapagliflozin in the heart failure with mildly reduced or preserved ejection (DELIVER) trial randomly assigned 6,263 patients with HF and left ventricular ejection fraction (LVEF) ≥ 40% to receive either dapagliflozin or placebo (Solomon et al., 2022). Dapagliflozin demonstrated a significant reduction in the risk of worsening HF or cardiovascular death (CVD) (Solomon et al., 2022).

The prospective comparison of ARNI with ARB [angiotensin receptor blockers] global outcomes in HF with the preserved ejection fraction (PARAGON-HF) trial randomly assigned 4,796 patients with symptomatic HF and LVEF ≥45% to receive either sacubitril–valsartan or valsartan alone (Solomon et al., 2019). The sacubitril–valsartan regimen demonstrated a non-statistically significant reduction in death from cardiovascular causes or hospitalization for HF, compared to the placebo (Solomon et al., 2019). However, a re-analysis of the results of this trial suggested a statistically significant difference (Felker et al., 2021).

Similarly, the Prospective comparison of ARNI with ARB Given following stabiLization In DEcompensated HFpEF (PARAGLIDE-HF) trial randomly assigned 466 patients with LVEF ≥ 40% stabilized after a worsening HF event to receive either sacubitril–valsartan or valsartan alone (Mentz et al., 2023). The sacubitril–valsartan regimen led to a statistically significant reduction in pro-B-type natriuretic peptide and a potential clinical benefit compared with valsartan, with fewer cardiovascular and renal events (Mentz et al., 2023).

The recent pooled analysis of all participants in the PARAGLIDE-HF and PARAGON-HF trials showed a significant reduction in the composite of total worsening HF events and CVD with sacubitril–valsartan compared to valsartan alone (Vaduganathan et al., 2023).

Although both dapagliflozin and sacubitril–valsartan can be attractive therapeutic options for patients with HFmrEF or HFpEF, a key aspect of treatment plans remains the associated cost. A recent study from our group compared dapagliflozin with the sacubitril–valsartan regimen in HFrEF patients and identified a monetary benefit for dapagliflozin over sacubitril–valsartan (Arbel et al., 2021). The aim of the present study is to compare the cost-per-outcome implications of prescribing dapagliflozin *versus* sacubitril–valsartan to prevent HF events and CVD in patients with HFmrEF or HFpEF.

## 2 Methods

### 2.1 Data sources for drug efficacy

Outcome data for dapagliflozin and sacubitril–valsartan were extracted from the DELIVER trial and pooled analysis of the PARAGLIDE-HF and PARAGON-HF trials, respectively (Solomon et al., 2022; Vaduganathan et al., 2023).

### 2.2 Outcome measures

The primary outcome was the cost needed to treat (CNT) to prevent one event of the composite outcome of total HF hospitalizations and CVD. CNT was also estimated for the prevention of one event of all-cause mortality. The analysis was performed from the perspective of healthcare payers in the United States.

### 2.3 Cost needed to treat analysis

The number of preventable hospitalizations for HF and CVD achievable with dapagliflozin or sacubitril–valsartan was estimated by dividing the predefined maximum available budget by the CNT to prevent one event. The budget limit, $735 million, was set as the United States’ threshold suggested by the Institute for Clinical and Economic Review (ICER) (Institute for Clinical and Economic Review, 2023). The CNT was calculated by multiplying the aNNT by the annual treatment cost (Mayne et al., 2006). Drug costs were calculated as 75% of the US National Average Drug Acquisition Cost (NADAC), extracted in July 2023 (Medicaid.gov, 2023).

### 2.4 Annualized number needed to treat analysis

The aNNT was calculated as one divided by the annualized absolute risk reduction (aARR), the absolute difference between the annualized absolute risk (aAR) in the control and treatment arms. The aAR of treatments was calculated by dividing the number of events in each study arm by patient-years of treatment (Mayne et al., 2006).

### 2.5 Scenario analysis

To evaluate the robustness of CNT results and mitigate differences between the baseline risk of the randomized controlled trial (RCT) populations, we performed one-way sensitivity analysis on parameters that may affect the NNT and CNT figures (Mendes et al., 2017): the risk of events in the control arm of the RCTs and the annual costs of the compared interventions.

### 2.6 Sensitivity analysis

To mitigate the differences in the risk of HF events in the RCTs, we simulated the effect of each drug while using each of the other drug control arms’ event rates. For the sensitivity analysis of the cost of therapy, we used the full NADAC price as the upper bound and 50% of the NADAC price as the lower bound, as recommended for use in US cost-effectiveness analyses (Levy et al., 2018).

## 3 Results

### 3.1 Patient populations

A total of 11,525 patients were included in the DELIVER and pooled analysis of the PARAGLIDE-HF and PARAGON-HF trials. The baseline characteristics of both trial participants are detailed in Table 1. The median follow-up time was longer in the pooled analysis of PARAGLIDE-HF and PARAGON-HF (2.8 years) compared to that of DELIVER (2.3 years).

**TABLE 1:** Baseline characteristics.

	Deliver N = 6,263		Paragon-HF N = 4,822		Paraglide-HF N = 466	
	Dapagliflozin (N = 3,131)	Placebo (N = 3,132)	Sacubitril–valsartan (N = 2,407)	Valsartan (N = 2,389)	Sacubitril–valsartan (N = 233)	Valsartan (N = 233)
**Age (years)**	71.8 ± 9.6	71.5 ± 9.5	72.7 ± 8.3	72.8 ± 8.5	71.0 (61.0-78.0)	72.0 (62.0-79.0)
**Female, no. (%)**	1,364 (43.6)	1,383 (44.2)	1,241 (51.6)	1,238 (51.8)	121 (51.9)	121 (51.9)
**Race, no. (%)**						
**Asian**	630 (20.1)	644 (20.6)	297 (12.3)	310 (13.0)	3 (1.3)	3 (1.3)
**Black**	81 (2.6)	78 (2.5)	52 (2.2)	50 (2.1)	50 (21.5)	52 (22.3)
**White**	2,214 (70.7)	2,225 (71.0)	1,963 (81.6)	1,944 (81.4)	176 (75.5)	176 (75.5)
**NYHA class no. (%)**						
**II**	2,225 (71.0)	2,399 (76.6)	1,866 (77.5)	1,840 (77.0)	102 (43.8)	101 (43.3)
**III**	807 (25.8)	724 (23.1)	458 (19.0)	474 (19.8)	117 (50.2)	112 (48.1)
**IV**	10 (0.3)	8 (0.3)	8 (0.3)	11 (0.5)	4 (1.7)	10 (4.3)
**LVEF, mean**	54.0 ± 8.6	54.3 ± 8.9	57.6 ± 7.8	57.5 ± 8.0	55.2 ± 8.06	55.7 ± 8.07
**Diabetes no. (%)**	1401 (44.7)	1405 (44.9)	1046 (43.5)	1016 (42.5)	107 (45.9)	119 (51.1)
**Hypertension no. (%)**	2,755 (88.0)	2,798 (89.3)	2,304 (95.7)	2,280 (95.4)	228 (97.9)	219 (94.0)
**eGFR**	61 ± 19	61 ± 19	63 ± 19	62 ± 19	47.4 (36.4-62.2)	51.1 (39.4-64.8)
**mL/min/** **1.73** **m** ^**2**^						

eGFR, estimated glomerular filtration rate; LVEF, left ventricular ejection fraction; NYHA, New York Heart Association.

### 3.2 Annualized number needed to treat and cost needed to treat

**FIGURE 1:**
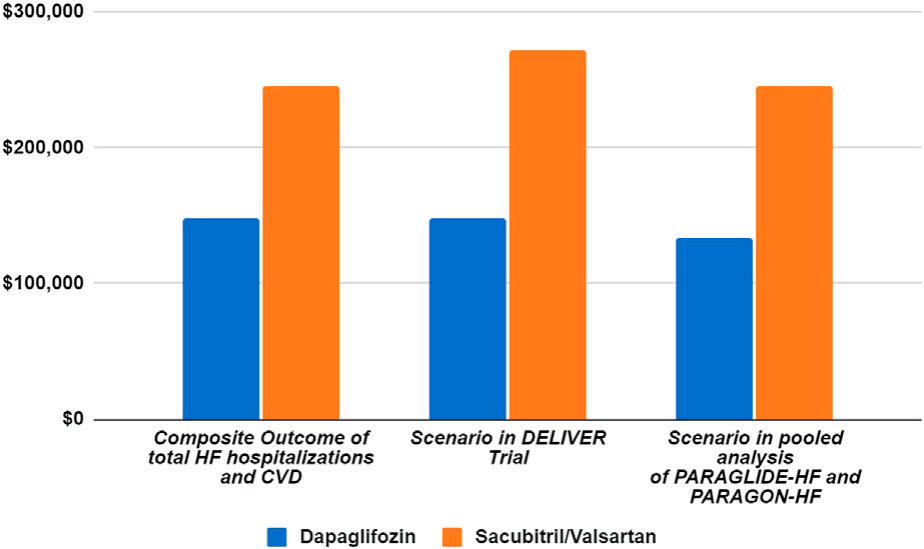
Scenario analysis of cost needed to treat based on the DELIVER and pooled analysis of PARAGLIDE-HF and PARAGON-HF trials.

The step-by-step calculations of the aNNT and CNT are outlined in Table 2. The annual drug costs are $4,951.57 for dapagliflozin and $5,576.06 for sacubitril–valsartan. The CNT to prevent one event of total worsening HF events and CVD (composite outcome) was $148,547.13 ($103,982.99–306,997.39) for dapagliflozin and $245,346.77 ($139,401.58–1,734,155.60) for sacubitril–valsartan (Figure 1).

**TABLE 2:** Step by step calculations of the number and cost needed to treat to prevent total worsening heart failure events and cardiovascular death with dapagliflozin vs sacubitril–valsartan.

Parameter	Dapagliflozin	Sacubitril–valsartan
Number of patients in the control arm	3,132	2,622
Follow-up (years)	2.3	2.8
Patient-years of therapy in the control arm	7,203.6	7,341.6
Number of events in the control arm	1,057	1,181
Annualized event rate in the control arm (%)	14.67	16.09
Number of patients in the intervention arm	3,131	2,640
Patient-years of therapy in intervention arm	7,201.3	7,392
Annualized event rate in the intervention arm, % (95% CI)	11.30 (9.83%–13.06%)	13.83 (12.06%–15.76%)
Annualized absolute event rate reduction, % (95% CI)	3.37 (1.61%–4.84%)	2.25 (0.32%–4.02%)
Annualized number needed to treat (95% CI)	30 (21–62)	44 (25–311)
Annual drug cost (US)	$ 4,951.57	$ 5,576.06
Cost needed to treat to prevent one event (95% CI)	$ 148,547.13 ($ 103,983–$ 306,997.39)	$ 245,346.77 ($ 139,401.58–$ 1,734,155.60)

CI, confidence internal; US, United States.

The CNT results of all-cause mortality are given in Table 3. Dapagliflozin had a lower CNT compared to a sacubitril–valsartan regimen to prevent one event of all-cause mortality: $1,128,958.15 ($401,077.24–∞) vs $2,185,816.71 ($ 607,790.87–∞).

**TABLE 3:** Cost needed to treat to prevent all-cause mortality with dapagliflozin vs sacubitril-valsartan.

	Dapagliflozin	Sacubitril–valsartan
Annualized event rate in the control arm (%)	7.30	5.11
Annualized event rate in the intervention arm, % (95% CI)	6.86 (6.06%–7.81%)	4.85 (4.19%–5.62%)
Annualized absolute event rate reduction, % (95% CI)	0.44 (0%–1.24%)	0.26 (0%–0.92%)
Annualized number needed to treat (95% CI)	228 (81–∞)	392 (109–∞)
All-cause mortality (95% CI)	$ 1,128,958.15 ($ 401,077.24–∞)	$ 2,185,816.71 ($ 607,790.87–∞)

CI, confidence interval.

### 3.3 Scenario analysis

Table 4 details the results of the sensitivity analysis performed by simulating the use of different annualized event rates in the control arm, according to the event rates in each of the trials.

**TABLE 4:** Results of simulating the effect of interventions in the randomized controlled trials.

	Annualized absolute risk	CNT for dapagliflozin	CNT for sacubitril–valsartan
Simulation of the annualized event rate in the RCT control group	14.67% (As in DELIVER)	Base case: $ 148,547.13 ($ 103,983–$ 306,997.39)	$ 273,226.94 ($ 150,553.62–$ 1,901,436.46)
	16.09% (as in pooled PARAGLIDE-HF and PARAGON-HF)	$ 133,692.39 ($ 94,079.83–$ 282,239.49)	Base case: $ 245,346.77 ($ 139,401.58–$ 1,734,155.60)

CNT, cost needed to treat; RCT, randomized controlled trial.

### 3.4 Sensitivity analysis

Table 5 and Figure 1 detail the results of the sensitivity analysis with different prices from the United States. If the annual predefined budget of $735 million was allocated entirely for the prevention of total HF hospitalizations and CVD, a higher number of events would be prevented using dapagliflozin (3,710 events, [95% CI: 1,795–5,301]), as compared to sacubitril–valsartan (2,246 events, [95% CI: 317–3,954] when utilizing the US high-cost estimate. We performed an additional sensitivity analysis using the reported event rate per 100 patient-years in the original studies, yielding similar CNT values to those derived from the reported rate ratio (Table 6).

**TABLE 5:** Sensitivity analysis: avoided heart failure and cardiovascular events in low- and high-cost estimates of dapagliflozin and sacubitril-valsartan.

Price estimate	US (low estimate)		US (high estimate)	
Treatment	Dapagliflozin	Sacubitril–valsartan	Dapagliflozin	Sacubitril–valsartan
Annual cost	$ 3,301.05	$ 3,717.38	$ 6,602.09	$ 7,434.74
Annual budget	$ 735,000,000			
CNT (95% CI)	$ 99,031.42 ($ 69,321.99 - $ 204,664.94)	$ 163,565 ($ 92,934 - $ 1,156,104)	$ 198,062.83 ($ 138,643.98 - $ 409,329.86)	$ 327,129 ($ 185,869 - $ 2,312,707)
Prevented events within the budget, N (95% CI)	7,421 (3,591–10,602)	4,498 (635–7,908)	3,710 (1,795–5,301)	2,246 (317–3,954)

CI, confidence interval; CNT, cost needed to treat; US, United States.

**TABLE 6:** Sensitivity analysis adopting the reported event rate per 100 patient-years versus the reported rate ratio in the randomized trials.

	Dapagliflozin		Sacubitril–valsartan	
	Rate ratio	Event rate (100 patient-years)	Rate ratio	Event rate (100 patient-years)
aAR in the control arm	14.67%	15.30%	16.09%	16.80%
aAR in the intervention arm	11.30%	11.80%	13.83%	14.50%
aARR	3.37%	3.50%	2.25%	2.30%
aNNT	30	29	44	43
CNT	$ 148,547.13	$ 143,595.53	$ 245,346.77	$ 239,770.71

aAR, annualized absolute risk; aARR, annualized absolute risk reduction; aNNT, annualized number needed to treat; CNT: cost needed to treat.

## 4 Discussion

In this cost–benefit study, leveraging data from the DELIVER, PARAGLIDE-HF, and PARAGON-HF trials, we demonstrated that dapagliflozin provides a better monetary value compared to sacubitril–valsartan for preventing the composite outcome of total worsening HF events and CVD and all-cause mortality as an individual outcome among patients with HFpEF.

As the population ages, the escalating prominence of HFpEF as a pivotal public health concern is anticipated (Tsao et al., 2023). This is associated with a cost that is estimated to reach $69.8 billion in annual healthcare spending by 2030 (Clark and Velazquez, 2020).

The DELIVER trial randomized patients with HFmrEF or HFpEF to either dapagliflozin or placebo. Over a median of 2.3 years, a statistically significant reduction in the primary composite outcome of HF hospitalization or CVD was found with dapagliflozin compared to the placebo (Solomon et al., 2022). The PARAGON-HF trial included patients with a similar clinical profile and randomized them to receive sacubitril–valsartan or valsartan alone. The trial narrowly missed statistical significance for its primary endpoint of a composite of total hospitalizations for HF and death from cardiovascular causes (Solomon et al., 2019). Similarly, the PARAGLIDE-HF trial showed that sacubitril–valsartan led to a greater reduction in plasma NT-proBNP levels compared to valsartan alone. However, the trial was not adequately powered to assess clinical outcomes (Mentz et al., 2023). Hence, Vaduganathan et al. (2023) performed a pooled analysis of PARAGLIDE-HF and PARAGON-HF trials; compared with valsartan, sacubitril–valsartan significantly reduced worsening HF events and CVD in the pooled analysis of all participants (Vaduganathan et al., 2023).

The 2023 Focused Update of the 2021 European Society of Cardiology (ESC) and 2022 American Heart Association/American College of Cardiology/Heart Failure Society of America (AHA/ACC/HFSA) guidelines for HF provide a Class 1 and Class 2a recommendation for SGLT2Is and Class 2b for ARNI for patients with HFmrEF and HFpEF (Heidenreich et al., 2022; McDonagh et al., 2023).

A recent meta-analysis of 13 studies with a total of 29,875 HF patients with LVEF >40% demonstrated that a quadruple-agent combination of SGLT2I, ARNI, beta blocker, and a mineralocorticoid receptor antagonist provides the largest reduction in the risk of CVD and HF hospitalization, largely attributed to the effect of the triple combination of SGLT2I, ARNI, and mineralocorticoid receptor antagonist. The benefit was more pronounced in HFmrEF patients (Zafeiropoulos et al., 2023). A combined SGLT2I and sacubitril–valsartan regimen has been approved and recommended for HFrEF (McDonagh et al., 2021), but its practice is not well-established for HFpEF patients. The high cost is likely to be a major limitation, serving as a barrier to prescribing the conjunctive regimen for HF patients (Luo et al., 2020).

Variances in the pharmacological mechanisms of action of dapagliflozin and sacubitril–valsartan may influence the reported differences in their clinical outcomes. The basic pharmacodynamic effect of SGLT2Is is the inhibition of SGLT2 in the proximal tubule of the nephron, leading to decreased glucose reabsorption into the blood and thus inducing a hypoglycemic effect (Vallon and Verma, 2021; Packer, 2022; 2023). However, these medications exert numerous other pharmacological properties that may contribute to their therapeutic benefits (Mustroph et al., 2018; Byrne et al., 2020; Quagliariello et al., 2021; Vallon and Verma, 2021; Koufakis et al., 2022; Packer, 2022; 2023). For example, dapagliflozin has been suggested to suppress neurohormonal activation, improve systolic function, and decrease the incidence of cardiac arrhythmias in patients with HF (Koufakis et al., 2022). Similarly, empagliflozin has been shown to inhibit the function of Ca^2+^/calmodulin-dependent kinase II, leading to improved myocardial contractility and a reduction of arrhythmias among HF patients (Mustroph et al., 2018). Additionally, empagliflozin was reported to inhibit the activity of the nucleotide-binding domain-like receptor protein 3 (NLRP3)-associated cellular pathways, resulting in a significant increase in left ventricular fractional shortening and ejection fraction and an overall improvement in cardiac function (Byrne et al., 2020; Quagliariello et al., 2021). It is worth noting in this regard that many natural compounds, such as resveratrol, possess some of the pharmacological properties of gliflozins, including potent inhibition of the NLRP3 inflammasome (Cocetta et al., 2021) and, therefore, may become a useful adjuvant treatment of cardiovascular disease (Sung and Dyck, 2015; Bonnefont-Rousselot, 2016; Raj et al., 2021) and other disorders (Cocetta et al., 2021; Amini et al., 2023). As for ARNI, these medications not only confer the regular, well-established pharmacological outcomes of angiotensin receptor blockers (such as valsartan) but also combine the therapeutic effects deriving from the inhibition of the enzyme neprilysin by sacubitril (Hubers and Brown, 2016; Velazquez et al., 2019; Pascual-Figal et al., 2021; Abdin et al., 2022). Neprilysin degrades atrial and brain natriuretic peptides, bradykinin, and other vasoactive peptides; its inhibition by sacubitril leads to a prominent vasodilatory effect and additional positive renal and cardiovascular outcomes (Hubers and Brown, 2016; Velazquez et al., 2019; Pascual-Figal et al., 2021; Abdin et al., 2022).

A European health-economic analysis of the DELIVER trial found that the addition of dapagliflozin to a standard of care is very likely cost-effective for HFmrEF or HFpEF in several European countries (Booth et al., 2023). Dapagliflozin treatment was predicted to increase quality-adjusted life years (QALYs) and life-years by 0.231 and 0.354, respectively, and prolong the time spent in the best quartile of the Kansas City Cardiomyopathy Questionnaire total symptom score (KCCQ-TSS) by 4.2 months. The incremental cost-effectiveness ratios were £7,761, €9,540, and €5343/QALY in the United Kingdom, Germany, and Spain, respectively (Booth et al., 2023). Similarly, Tang and Sang. (2023) performed a cost-utility analysis based on the DELIVER study and the national statistical database. The study showed that the adjunct use of dapagliflozin to standard of care among patients with HFpEF or HFmrEF was cost-effective in China at a willingness-to-pay value of $12,652.5/QALY (Tang and Sang, 2023). Most recently, Cohen et al. (2023) performed an economic evaluation using a simulation model of US adults with HFpEF who meet the eligibility criteria of the EMPEROR-Preserved or DELIVER trials. They found that the addition of an SGLT2I to the standard of care increased quality-adjusted survival by 0.19 QALYs at an increased cost of $26,300 compared with standard of care. The resulting incremental cost-effectiveness ratio was $141,200 per QALY gained, which is of intermediate or low economic value compared with standard of care in HFpEF (Cohen et al., 2023).

There has been limited data on the cost-effectiveness of sacubitril–valsartan in patients with HFpEF. Recently, Wang et al. (2023) investigated the cost-effectiveness of sacubitril–valsartan as an alternative to valsartan in Chinese patients with HFpEF. They found the ICER for sacubitril–valsartan to be $49,019/QALY ($46,610/life-year), higher than the willingness-to-pay threshold and hence not cost-effective (Wang et al., 2023). However, a recent economic evaluation using participant-level data from the PARADIGM-HF and PARAGON-HF trials (*n* = 13,264) found sacubitril–valsartan to be more cost-effective at lower EF ranges with a high economic value for patients with HFrEF or HFmEF (EF ≤ 50%) and at least of intermediate value to an EF ≤ 60% compared with renin–angiotensin system inhibitors. Only in those with EFs of 45% or greater did sacubitril–valsartan yield an incremental cost-effectiveness ratio of $127,172 per QALY gained (Bhatt et al., 2023).

Although a growing body of evidence supports the role of SGLT2Is and ARNI in reducing HF hospitalization and CVD among HF patients, their use is still limited in clinical practice partially due to their cost. The unaffordability of pharmacotherapy due to high cost is a major reason for nonadherence to prescribed medications (De Avila et al., 2021; Simon et al., 2021; Dusetzina et al., 2023). Our analysis attempts to provide some cost-per-outcome insight when prescribing dapagliflozin and sacubitril–valsartan. To the best of our knowledge, this is the first cost-per-outcome comparison between dapagliflozin and sacubitril–valsartan in patients with HFmrEF and HFpEF. Future studies are needed to confirm these findings.

### 4.1 Limitations

Our study has several limitations that warrant consideration. First, despite the similarity in patient populations across both trials, the presence of apparent differences between them poses a limitation to our analysis. We attempted to mitigate this through sensitivity analysis by simulating the effects of each drug within each RCT. Second, it is important to note that our analysis does not substitute the need for a comprehensive cost-effectiveness assessment in relation to QALY and potential cost savings associated with preventing HF hospitalization. Although such an assessment is necessary, it remains unavailable due to the recent completion of DELIVER and the pooled analysis of PARAGLIDE-HF and PARAGON-HF trials. A third limitation pertains to the reliance on aNNT estimates in our CNT figure, which has its own restrictions (Saver and Lewis, 2019). However, NNT has been found to be useful for assisting decision-making in many clinical settings (Mendes et al., 2017; Saver and Lewis, 2019) and is required by the Consolidated Standards of Reporting Trials statement to be reported in RCT publications (Moher et al., 2012). Moreover, the annualization of NNT for comparing RCTs and therapies is a method employed in previous studies (Chew et al., 2009; Fonarow et al., 2011). Lastly, our findings are based on the published results of only a limited number of HFpEF patients which may restrict the generalizability of our analysis to a broader HFpEF population.

## 5 Conclusion

In analyzing data from the DELIVER and the pooled analysis of PARAGLIDE-HF and PARAGON-HF trials, the CNT to prevent HF hospitalizations and CVD was lower for dapagliflozin compared to sacubitril–valsartan for HFmrEF and HFpEF patients.

## Data Availability

The original contributions presented in the study are included in the article/Supplementary material; further inquiries can be directed to the corresponding authors.

## References

[B1] Abdin A. Schulz M. Riemer U. Hadëri B. Wachter R. Laufs U. (2022). Sacubitril/valsartan in heart failure: efficacy and safety in and outside clinical trials. Esc. Heart Fail 9, 3737–3750. 10.1002/ehf2.14097 35921043 PMC9773772

[B2] Amini P. Moazamiyanfar R. Dakkali M. S. Khani A. Jafarzadeh E. Mouludi K. (2023). Resveratrol in cancer therapy: from stimulation of genomic stability to adjuvant cancer therapy: a comprehensive review. Curr. Top. Med. Chem. 23, 629–648. 10.2174/1568026623666221014152759 36239730

[B3] Arbel R. Aboalhasan E. Hammerman A. Azuri J. (2021). Dapagliflozin vs. sacubitril-valsartan for prevention of heart failure events in non-diabetic patients with reduced ejection fraction: a cost per outcome analysis. Eur. J. Prev. Cardiol. 28, 1665–1669. 10.1093/eurjpc/zwaa136 33624086

[B4] Bhatt A. S. Vaduganathan M. Claggett B. L. Fonarow G. C. Packer M. Pfeffer M. A. (2023). Health and economic evaluation of sacubitril-valsartan for heart failure management. JAMA Cardiol. 8, 1041–1048. 10.1001/jamacardio.2023.3216 37755814 PMC10534998

[B5] Bonnefont-Rousselot D. (2016). Resveratrol and cardiovascular diseases. Nutrients 8, 250. 10.3390/nu8050250 27144581 PMC4882663

[B6] Booth D. Davis J. A. McEwan P. Solomon S. D. McMurray J. J. V. De Boer R. A. (2023). The cost‐effectiveness of dapagliflozin in heart failure with preserved or mildly reduced ejection fraction: a European health‐economic analysis of the DELIVER trial. Eur. J. Heart Fail 25, 1386–1395. 10.1002/ejhf.2940 37344985

[B7] Byrne N. J. Matsumura N. Maayah Z. H. Ferdaoussi M. Takahara S. Darwesh A. M. (2020). Empagliflozin blunts worsening cardiac dysfunction associated with reduced NLRP3 (Nucleotide-Binding domain-like receptor protein 3) inflammasome activation in heart failure. Circ. Heart Fail 13, e006277. 10.1161/CIRCHEARTFAILURE.119.006277 31957470

[B8] Chew D. P. Huynh L. T. Liew D. Astley C. Soman A. Brieger D. (2009). Potential survival gains in the treatment of myocardial infarction. Heart 95, 1844–1850. 10.1136/hrt.2009.174276 19666459

[B9] Clark K. A. A. Velazquez E. J. (2020). Heart failure with preserved ejection fraction: time for a reset. JAMA 324, 1506–1508. 10.1001/jama.2020.15566 33079136

[B10] Cocetta V. Quagliariello V. Fiorica F. Berretta M. Montopoli M. (2021). Resveratrol as chemosensitizer agent: state of art and future perspectives. Int. J. Mol. Sci. 22, 2049. 10.3390/ijms22042049 33669559 PMC7922064

[B11] Cohen L. P. Isaza N. Hernandez I. Lewis G. D. Ho J. E. Fonarow G. C. (2023). Cost-effectiveness of sodium-glucose cotransporter-2 inhibitors for the treatment of heart failure with preserved ejection fraction. JAMA Cardiol. 8, 419–428. 10.1001/jamacardio.2023.0077 36870047 PMC9985815

[B12] De Avila J. L. Meltzer D. O. Zhang J. X. (2021). Prevalence and persistence of cost-related medication nonadherence among medicare beneficiaries at high risk of hospitalization. JAMA Netw. Open 4, e210498. 10.1001/jamanetworkopen.2021.0498 33656528 PMC7930921

[B13] Dusetzina S. B. Besaw R. J. Whitmore C. C. Mattingly T. J. Sinaiko A. D. Keating N. L. (2023). Cost-related medication nonadherence and desire for medication cost information among adults aged 65 Years and older in the US in 2022. JAMA Netw. Open 6, e2314211. 10.1001/jamanetworkopen.2023.14211 37200029 PMC10196872

[B14] Felker G. M. Butler J. Januzzi J. L. Desai A. S. McMurray J. J. V. Solomon S. D. (2021). Probabilistic readjudication of heart failure hospitalization events in the PARAGON-HF study. Circulation 143, 2316–2318. 10.1161/CIRCULATIONAHA.121.054496 34097449

[B15] Fonarow G. C. Yancy C. W. Hernandez A. F. Peterson E. D. Spertus J. A. Heidenreich P. A. (2011). Potential impact of optimal implementation of evidence-based heart failure therapies on mortality. Am. Heart J. 161, 1024–1030. 10.1016/j.ahj.2011.01.027 21641346

[B16] Heidenreich P. A. Bozkurt B. Aguilar D. Allen L. A. Byun J. J. Colvin M. M. (2022). 2022 AHA/ACC/HFSA guideline for the management of heart failure: a report of the American College of Cardiology/American heart association joint committee on clinical practice guidelines. Circulation 145, e895–e1032. 10.1161/CIR.0000000000001063 35363499

[B17] Hubers S. A. Brown N. J. (2016). Combined angiotensin receptor antagonism and neprilysin inhibition. Circulation 133, 1115–1124. 10.1161/CIRCULATIONAHA.115.018622 26976916 PMC4800749

[B18] Institute for Clinical and Economic Review (2023). Value assessment framework. Available at: https://icer.org/wp-content/uploads/2023/10/ICER_2023_VAF_For-Publication_101723.pdf (Accessed December 8, 2023).

[B19] Koufakis T. Giannakoulas G. Zebekakis P. Kotsa K. (2022). The effect of dapagliflozin on ventricular arrhythmias, cardiac arrest, or sudden death in people with heart failure: a tick in another box for sodium-glucose cotransporter 2 inhibitors. Expert Opin. Pharmacother. 23, 321–325. 10.1080/14656566.2021.2003329 34761713

[B20] Levy J. Rosenberg M. Vanness D. (2018). A transparent and consistent approach to assess US outpatient drug costs for use in cost-effectiveness analyses. Value Health 21, 677–684. 10.1016/j.jval.2017.06.013 29909872 PMC6394851

[B21] Luo J. Feldman R. Rothenberger S. D. Hernandez I. Gellad W. F. (2020). Coverage, formulary restrictions, and out-of-pocket costs for sodium-glucose cotransporter 2 inhibitors and glucagon-like peptide 1 receptor agonists in the medicare Part D program. JAMA Netw. Open 3, e2020969. 10.1001/jamanetworkopen.2020.20969 33057641 PMC7563069

[B22] Mayne T. J. Whalen E. Vu A. (2006). Annualized was found better than absolute risk reduction in the calculation of number needed to treat in chronic conditions. J. Clin. Epidemiol. 59, 217–223. 10.1016/j.jclinepi.2005.07.006 16488351

[B23] McDonagh T. A. Metra M. Adamo M. Gardner R. S. Baumbach A. Böhm M. (2021). 2021 ESC Guidelines for the diagnosis and treatment of acute and chronic heart failure. Eur. Heart J. 42, 3599–3726. 10.1093/eurheartj/ehab368 34447992

[B24] McDonagh T. A. Metra M. Adamo M. Gardner R. S. Baumbach A. Böhm M. (2023). 2023 Focused Update of the 2021 ESC Guidelines for the diagnosis and treatment of acute and chronic heart failure. Eur. Heart J. 44, 3627–3639. 10.1093/eurheartj/ehad195 37622666

[B25] McMurray J. J. V. Jackson A. M. Lam C. S. P. Redfield M. M. Anand I. S. Ge J. (2020). Effects of sacubitril-valsartan versus valsartan in women compared with men with heart failure and preserved ejection fraction: insights from PARAGON-HF. Circulation 141, 338–351. 10.1161/CIRCULATIONAHA.119.044491 31736337 PMC12611550

[B26] McMurray J. J. V. Packer M. Desai A. S. Gong J. Lefkowitz M. P. Rizkala A. R. (2014). Angiotensin–neprilysin inhibition versus enalapril in heart failure. N. Engl. J. Med. 371, 993–1004. 10.1056/NEJMoa1409077 25176015

[B27] McMurray J. J. V. Solomon S. D. Inzucchi S. E. Køber L. Kosiborod M. N. Martinez F. A. (2019). Dapagliflozin in patients with heart failure and reduced ejection fraction. N. Engl. J. Med. 381, 1995–2008. 10.1056/NEJMoa1911303 31535829

[B28] Medicaid.gov (2023). Pharmacy pricing. Available at: https://www.medicaid.gov/medicaid/prescription-drugs/pharmacy-pricing/index.html (Accessed December 8, 2023).

[B29] Mendes D. Alves C. Batel-Marques F. (2017). Number needed to treat (NNT) in clinical literature: an appraisal. BMC Med. 15, 112. 10.1186/s12916-017-0875-8 28571585 PMC5455127

[B30] Mentz R. J. Ward J. H. Hernandez A. F. Lepage S. Morrow D. A. Sarwat S. (2023). Angiotensin-neprilysin inhibition in patients with mildly reduced or preserved ejection fraction and worsening heart failure. J. Am. Coll. Cardiol. 82, 1–12. 10.1016/j.jacc.2023.04.019 37212758

[B31] Moher D. Hopewell S. Schulz K. F. Montori V. Gøtzsche P. C. Devereaux P. J. (2012). CONSORT 2010 explanation and elaboration: updated guidelines for reporting parallel group randomised trials. Int. J. Surg. 10, 28–55. 10.1016/j.ijsu.2011.10.001 22036893

[B32] Mustroph J. Wagemann O. Lücht C. M. Trum M. Hammer K. P. Sag C. M. (2018). Empagliflozin reduces Ca/calmodulin-dependent kinase II activity in isolated ventricular cardiomyocytes. Esc. Heart Fail 5, 642–648. 10.1002/ehf2.12336 30117720 PMC6073019

[B33] Packer M. (2022). Critical reanalysis of the mechanisms underlying the cardiorenal benefits of SGLT2 inhibitors and reaffirmation of the nutrient deprivation signaling/autophagy hypothesis. Circulation 146, 1383–1405. 10.1161/CIRCULATIONAHA.122.061732 36315602 PMC9624240

[B34] Packer M. (2023). SGLT2 inhibitors: role in protective reprogramming of cardiac nutrient transport and metabolism. Nat. Rev. Cardiol. 20, 443–462. 10.1038/s41569-022-00824-4 36609604

[B35] Packer M. Anker S. D. Butler J. Filippatos G. Pocock S. J. Carson P. (2020). Cardiovascular and renal outcomes with empagliflozin in heart failure. N. Engl. J. Med. 383, 1413–1424. 10.1056/NEJMoa2022190 32865377

[B36] Pascual-Figal D. Bayés-Genis A. Beltrán-Troncoso P. Caravaca-Pérez P. Conde-Martel A. Crespo-Leiro M. G. (2021). Sacubitril-valsartan, clinical benefits and related mechanisms of action in heart failure with reduced ejection fraction. A review. Front. Cardiovasc Med. 8, 754499. 10.3389/fcvm.2021.754499 34859070 PMC8631913

[B37] Pitt B. Pfeffer M. A. Assmann S. F. Boineau R. Anand I. S. Claggett B. (2014). Spironolactone for heart failure with preserved ejection fraction. N. Engl. J. Med. 370, 1383–1392. 10.1056/NEJMoa1313731 24716680

[B38] Quagliariello V. De Laurentiis M. Rea D. Barbieri A. Monti M. G. Carbone A. (2021). The SGLT-2 inhibitor empagliflozin improves myocardial strain, reduces cardiac fibrosis and pro-inflammatory cytokines in non-diabetic mice treated with doxorubicin. Cardiovasc Diabetol. 20, 150. 10.1186/s12933-021-01346-y 34301253 PMC8305868

[B39] Raj P. Thandapilly S. J. Wigle J. Zieroth S. Netticadan T. (2021). A comprehensive analysis of the efficacy of resveratrol in atherosclerotic cardiovascular disease, myocardial infarction and heart failure. Molecules 26, 6600. 10.3390/molecules26216600 34771008 PMC8587649

[B40] Saver J. L. Lewis R. J. (2019). Number needed to treat: conveying the likelihood of a therapeutic effect. JAMA 321, 798–799. 10.1001/jama.2018.21971 30730545

[B41] Simon S. T. Kini V. Levy A. E. Ho P. M. (2021). Medication adherence in cardiovascular medicine. BMJ 374, n1493. 10.1136/bmj.n1493 34380627

[B42] Solomon S. D. McMurray J. J. V. Anand I. S. Ge J. Lam C. S. P. Maggioni A. P. (2019). Angiotensin-neprilysin inhibition in heart failure with preserved ejection fraction. N. Engl. J. Med. 381, 1609–1620. 10.1056/NEJMoa1908655 31475794

[B43] Solomon S. D. McMurray J. J. V. Claggett B. de Boer R. A. DeMets D. Hernandez A. F. (2022). Dapagliflozin in heart failure with mildly reduced or preserved ejection fraction. N. Engl. J. Med. 387, 1089–1098. 10.1056/NEJMoa2206286 36027570

[B44] Sung M. M. Dyck J. R. B. (2015). Therapeutic potential of resveratrol in heart failure. Ann. N. Y. Acad. Sci. 1348, 32–45. 10.1111/nyas.12839 26205211

[B45] Tang Y. Sang H. (2023). Cost‐utility analysis of add‐on dapagliflozin in heart failure with preserved or mildly reduced ejection fraction. Esc. Heart Fail 10, 2524–2533. 10.1002/ehf2.14426 37290665 PMC10375078

[B46] Tsao C. W. Aday A. W. Almarzooq Z. I. Anderson C. A. M. Arora P. Avery C. L. (2023). Heart disease and stroke statistics—2023 update: a report from the American heart association. Circulation 147, e93–e621. 10.1161/CIR.0000000000001123 36695182 PMC12135016

[B47] Vaduganathan M. Mentz R. J. Claggett B. L. Miao Z. M. Kulac I. J. Ward J. H. (2023). Sacubitril/valsartan in heart failure with mildly reduced or preserved ejection fraction: a pre-specified participant-level pooled analysis of PARAGLIDE-HF and PARAGON-HF. Eur. Heart J. 44, 2982–2993. 10.1093/eurheartj/ehad344 37210743 PMC10424880

[B48] Vallon V. Verma S. (2021). Effects of SGLT2 inhibitors on kidney and cardiovascular function. Annu. Rev. Physiol. 83, 503–528. 10.1146/annurev-physiol-031620-095920 33197224 PMC8017904

[B49] Velazquez E. J. Morrow D. A. DeVore A. D. Duffy C. I. Ambrosy A. P. McCague K. (2019). Angiotensin–neprilysin inhibition in acute decompensated heart failure. N. Engl. J. Med. 380, 539–548. 10.1056/NEJMoa1812851 30415601

[B50] Wang Z. Lou Y. Wang Q. Sun M. Li X. Wang Y. (2023). Sacubitril/valsartan for heart failure with preserved ejection fraction: a cost-effectiveness analysis from the perspective of the Chinese healthcare system. Clin. Drug Investig. 43, 265–275. 10.1007/s40261-023-01249-8 36976423

[B51] Zafeiropoulos S. Farmakis I. T. Milioglou I. Doundoulakis I. Gorodeski E. Z. Konstantinides S. V. (2023). Pharmacological treatments in heart failure with mildly reduced and preserved ejection fraction. JACC Heart Fail. 10.1016/j.jchf.2023.07.014 37656079

